# Review of "Multiple Pregnancy: Epidemiology, Gestation & Perinatal Outcome. Second Edition"

**DOI:** 10.1186/1743-1050-2-12

**Published:** 2005-08-31

**Authors:** Edward J Hayes

**Affiliations:** 1 Department of Obstetrics and Gynecology, Thomas Jefferson University, Suite 400, Ben Franklin Building, Philadelphia, PA 19107, USA

Over the past two decades, an epidemic of multiple pregnancies has taken place in the developed world due to the widespread use of assisted reproductive technology. The general public has come to accept the phenomenon of higher order multiple as being a normal occurrence, expecting a good outcome for both the fetuses and the mother. However, physicians caring for these patients appreciate that these pregnancies have an increased number of complications both for the mother and fetus. The mother suffers substantial morbidity not just due to the risk and interventions of prematurity but also the increased incidence in medical complications. The fetuses are at jeopardy not just due to premature delivery, but also the increased rates of anomalies in multiples, and those complications uniquely associated with multiples, such as twin to twin transfusion syndrome. Clinicians involved in management of these high-risk pregnancies regrettably have had to rely on protocols based on expert opinion with unproven efficacy. These protocols may unintentionally place a mother and her unborn children at significant risk without achieving the desired benefit.

This book proves to be a valuable resource to those involved in all aspects of caring for multiple gestations. The book is divided into eleven main sections with a natural progression from first addressing the epidemiologic aspect of multiples and then centering on the individual patient, starting with etiology of multiples and following through to postpartum childhood concerns. This depth of information is the true strength of this resource, for it is not just a book of protocols, but rather it helps one to examine the causes of the current epidemic and suggests interventions to control this trend, while still allowing an individual practioner to optimize patient care.

The quality of the publication is excellent, with easy to read graphs, tables, and ultrasound images which actually are decipherable-a weakness in so many textbooks. The references are current, well documented, and relevant. What is most striking in this text is the quality of the illustrations. The editors give well-deserved special thanks to David Teplica for his contributions illustrating the phenomenon of mirror twins. These images allow the reader to better appreciate and comprehend this occurrence.

The first section of this volume is concerned primarily with epidemiologic and demographic trends in multiple gestations. This section is especially tailored to those individuals involved in public policy, and practioners taking care of the infertile patient. Throughout this segment it is apparent the immense impact assisted reproductive technologies has had on the growing incidence of multiples. Several regions are examined both in reference to the rate of multiples and their attempts to control this trend. The reporting of the Finnish randomized study will have an impact on any reader, and hopefully will change ones thinking about optimizing pregnancy rates through multiple embryo transfers.

The text also addresses the biologic considerations of multiple gestations. This begins with the causes of multiples we all recall from medical school, but then goes on to discuss the newer etiologies of multiples such as ART and current research in the field even examining the questionable link between folic acid and twins. Several subdivisions are devoted to iatrogenic twining, not just commenting on the overall increased incidence, but also the type of technology related to a particular zygosity types. For example, one section is devoted to the role of micromanipulation and the increased incidence of monozygotic twins with IVF. Development events seen in multiples are also discussed, not just the common issues, but also rare events, including complete hydatiform mole with coexistent twin. Of note, the segment on cloning is truly exceptional, for it explains this technology very clearly and goes on to discuss the debate over its use comparing it to the initial debate with IVF.

The prenatal diagnosis portion is devoted primarily to the role of ultrasound in the evaluation of multiple gestations. This begins with a stepwise approach to the diagnosis of chorionicity and amnionicity, including an interesting commentary by *S. Tong *discussing the possible role of the number of corpora lutea in determing zygosity. This transitions to the promising position that three-dimensional sonography may become a standard in the management of multiples, with text supported by superb images. However, a clinician involved in the care of multiples will find the content on aneuploidy most clinically relevant particulary when counseling the patients about risk of an anomalous fetus and the optimal diagnostic testing for the individual patient. The limitations of serum screening for trisomy 21 in this population are examined, as well as the role of nuchal transluceny as a screening test. The chapter by *Pergament *which addresses at what maternal age to offer invasive genetic testing to a mother of multiples is an essential part of patient care.

The segment of the text titled "Pregnancy management: maternal" is one of the most well balanced presentations of this difficult topic. For the author recognizes that the care of multiple gestations is inadequately studied and based on individual experience rather than randomized trials proving the therapeutic intervention is beneficial to the patient. Therefore throughout this segment the reader is informed of the basis of the clinical recommendation, whether there is strong evidence-based support for specific management recommendations or if this level of support is missing. An illustration of this is the discussion of surveillance of fetal well-being with respect to the routine use of antepartum surveillance and conveying that this has limited literature to support this intervention. There are also two excellent subdivisions of this section, covering hypertensive disorders and diabetes, which warrant special acknowledgement. The section on hypertensive disorders is exceptionally thorough, it not only addresses the routine associations but also covers the atypical presentation of pre-eclampsia in multiples and the significant relationship of acute fatty liver of pregnancy with triplets. The diabetic chapter is also unique for an obstetrical resource book in that through a thoughtful review of the literature the reader comes to appreciate that there is not a clear-cut association between gestational diabetes mellitus and multiples.

Those interventions specifically aimed at improving fetal outcomes in multiples is explored comprehensively in this text. This review investigates the spectrum of interuterine therapy addressing the current debate as well as techniques for multifetal pregnancy reduction through to the optimal invasive treatment of twin-twin transfusion syndrome, by referencing the best literature available at this time. A portion of this section addresses measures to reduce the incidence of preterm delivery, and although thoroughly analyzed, some of the conclusions are open to discussion, particularly with reference to interventions to inhibit preterm labor. The editors try to resolve this issue by presenting multiple views, but as seen through this text and throughout the literature the optimal treatment remains controversial.

The academic deliberations continue in the sections dealing with optimizing maternal and fetal outcome via route of delivery. Several opinions are presented in the debate of elective cesarean versus vaginal delivery of multiples, even having a chapter titled "Delivery of multiples: a dissenting view" which should be required reading for any individual involved in the delivery of multiples.

The content of this text also examines the multiple postpartum subjects encountered when caring for multiples. The maternal side is addressed from puerperal period commenting on postpartum hemorrhage to long term maternal issues such as incontinence and pelvic floor dysfunction. The neonatal discussion is also extensive not just addressing those issues related to prematurity but also unique aspects in caring for multiples such as the benefits of co-bedding the infants. This discussion is then followed by a section addressing childhood issues pertaining to multiples, particularly the association between multiples and cerebral palsy not just addressing the causative factors but also the familial adjustment issues and the effect this disorder has on the non-disabled multiple-birth children.

The final part of this text addressing clinical care of the individual patient is focused on familial issues. This section begins with the early part of pregnancy examining the psychological impact of multifetal pregnancy reduction progressing to post-natal issues such as the higher rate of child abuse in multiple birth families. A strong component of this is a wonderful discussion on the difficult topic of fetal or infant death which is encountered more frequently with multiples. This discussion centered on the bereavement period, and familial coping strategies and providing resources available to individuals who are going through this tragic event.

The concluding portion of this book examines the legal and ethical issues that are part of caring for multiples. It examines these issues both on an individual practioner level as well as a society trying to come to grips with the difficult choices that arise in the management of these patients.

Through this review it is apparent the broad scope this text covers, benefiting those who are involved with ART, as well as pediatricians caring for the adolescent multiples and all those care givers in between. The quality of this publication was superior, with only two areas which could have been improved. The first was excessive overlap. This happens in many multi-authored texts but there were several sections which could have been combined to allow for smoother flow and better transition with less repition, such as the multiple areas addressing the role of ART in the incidence of multiples. The other comment was some of the sections had theories which although academically interesting did not have the support required to be part of a reference text, this is evidenced by the discussion that the origin of monozygotic twins may depend mainly on a change in gonadotropin secretion acting on the preovulatory oocyte. These observations, however, should not defer anyone from acquiring this book, for overall it will prove to be an excellent resource for any care giver of multiples whether at the office level or involved in policy making trying to control this iatrogenic epidemic while maximizing patient outcome. Therefore, based on the prior observations, it is without reservation that I invite one to make this text a part of their personal library, for you will find it to be a valuable resource when caring for multiple gestations.

